# Effect of Low Positive End of Treatment Viral Load with Direct-Acting Antiviral Therapy on Sustained Virologic Response

**DOI:** 10.1155/2020/8815829

**Published:** 2020-07-27

**Authors:** Vabhave Pal, Nirupama Ancha, Jena Mann, Apurva A. Modi

**Affiliations:** ^1^Department of Internal Medicine, Texas Tech University Health Sciences Center at the Permian Basin, Odessa, TX, USA; ^2^Baylor University, Waco, TX, USA; ^3^Waco Gastroenterology Associates, Waco, TX, USA; ^4^Liver Consultants of Texas, Baylor Scott & White All-Saints Medical Center, Fort Worth, TX, USA

## Abstract

**Background:**

Direct-acting antivirals (DAAs) are highly effective treatments against hepatitis C virus (HCV), with sustained virologic response (SVR) rates of 93–100% against all genotypes. In most patients, viral load (VL) becomes undetectable four weeks into treatment, but rarely a low positive VL may be observed at the end of treatment (EOT). This study was conducted to determine the effect of low positive EOT VLs with DAA therapies on SVR at 12 and 24 weeks.

**Methods:**

A retrospective chart review was conducted from January 2014 to December 2018 on 1256 HCV patients of all genotypes (1–6) who had received DAA therapy at two large hepatology referral centers. Baseline demographic data, along with VL at week four, EOT, and SVR12/24 time points were collected for patients that had positive EOT VL. Treatment outcome for any patient with positive EOT VL was noted.

**Results:**

Eight out of 1256 patients treated with varying DAA therapies were observed to have low positive EOT VLs ranging from <15 to 235 IU/mL. One patient had a negative EOT VL, but 23 IU/mL at week four after EOT. All eight patients who had low positive EOT VLs and one patient who had a low positive VL at four weeks after EOT achieved SVR at weeks 12 and 24. One of the eight patients had cirrhosis. The majority of patients were genotype 1a.

**Conclusion:**

In the DAA treatment era, low levels of detectable HCV RNA at EOT does not predict treatment failure.

## 1. Introduction

Chronic hepatitis C impacts an estimated 70 million people worldwide [1]. The standard of care for treatment of the hepatitis C virus (HCV) is direct-acting antivirals (DAAs). Sustained virological response (SVR) is used as the primary efficacy endpoint for treatment, defined as HCV RNA levels below a designated threshold of quantification 24 weeks after the end of treatment (SVR24). More recently, SVR12 has been accepted as a valid efficacy endpoint because of its high rate of concordance with SVR24 [2]. Treatment with DAAs is associated with high rates of SVR along with minimal side effects [3].

During the previous era of interferon treatment for HCV, detectable viral load (VL) at end of treatment (EOT) negatively impacted the chance of achieving SVR [4, 5]. The significance of a low positive VL observed at EOT is unclear. We sought to determine the effect of EOT low positive VL with DAA therapies on SVR12 and SVR24. We hypothesized that most patients with low positive VL at EOT would still achieve SVR12 and SVR24.

## 2. Methods

### 2.1. Patients and Study Design

This was a retrospective chart review of 1256 consecutive patients who had received DAA therapy from January 2014 to December 2018 at two large hepatology referral centers in the Dallas-Fort Worth area. Eligible HCV patients of all genotypes (1–6) that received recommended DAA therapies were included. Data collected included demographics, treatment regimen, and relevant lab values at start of treatment. Quantitative HCV RNA levels were checked four weeks into therapy, EOT, and four weeks, 12 weeks, and 24 weeks after treatment.

HCV RNA levels were measured using the Roche COBAS TaqMan HCV test. The assay has a lower limit of quantification (LLOQ), defined as the lowest HCV RNA concentration that can be accurately quantified, of 15 IU/mL. Any value below 15 IU/mL was determined to be unquantifiable. Treatment outcome for any patient with positive VL at EOT or beyond was noted.

## 3. Results

Demographic, lab, and treatment characteristics of patients in the study are shown in Table 1. Eight of the nine patients were Caucasian, and the majority were female. 1248 patients in the study had HCV RNA < LLOQ at EOT and achieved SVR12.

Of the remaining eight patients who had low positive EOT VLs, all achieved SVR12 and SVR24. The EOT VL ranged from <15 to 235 IU/mL. The highest VL observed was 235 IU/mL in a patient with genotype 2 treated with sofosbuvir and ribavirin for 12 weeks. The majority of patients were genotype 1a, and one patient had cirrhosis. The various DAA treatment regimens in EOT positive patients included sofosbuvir/ledipasvir for 12 weeks (*n* = 4), sofosbuvir/simeprevir for 12 weeks (*n* = 1), sofosbuvir/ribavirin for 12 weeks (*n* = 1), sofosbuvir/velpatasvir/voxilaprevir for 12 weeks (*n* = 1), and glecaprevir/pibrentasvir for 8 weeks (*n* = 1).

One patient that received sofosbuvir/ledipasvir had a negative EOT VL, but 23 IU/mL at week four after EOT also achieved SVR12 and SVR24.

## 4. Discussion

This study evaluated the significance of EOT VL measurements in patients treated with various DAA regimens. When EOT VL is found to be positive, the question arises about extending treatment duration. The results of the study show that positive EOT VLs were not associated with treatment failure in patients treated with DAAs; hence, treatment duration need not be extended. The results also support the view that the utility of checking EOT VLs is low as all patients will likely achieve SVR.

Chronic hepatitis C infection develops with the loss of function of CD8 T-cells, known as T-cell exhaustion. Achieving SVR despite EOT positive VL can be explained by various hypotheses. The first is that DAA treatment leads to low levels of noninfectious virions unable to establish chronic infection, which are eventually cleared, leading to SVR [6, 7]. The second hypothesis argues that clearance of infected virions leads to a decline in T-cell exhaustion and simultaneous activation of cytotoxic T-cells that drive viral clearance [8–11]. The latter explanation is supported by research showing sustained and enhanced total T-cell immune response even after DAA therapy [12]. Third, it has been suggested that in patients who achieve SVR, viral suppression by DAAs results in an enhanced type I IFN response leading to higher activation of interferon-stimulated gene expression at EOT, resulting in elimination of residual virus [13]. Positive EOT VL may also be explained by laboratory contamination at the time the sample was analyzed.

The relationship between EOT low positive VL and SVR has been shown in a few studies. An analysis of patients treated with sofosbuvir and ledipasvir with HCV RNA above the LLOQ at EOT showed that all achieved SVR [14]. A retrospective study with 89 patients receiving sofosbuvir-based therapies showed that of the 6% of patients with EOT positive VL, all achieved SVR24 [15]. Another study of patients getting sofosbuvir-based therapy showed that rates of SVR12 among patients with undetectable EOT VL versus low-level viremia were similar [16]. The results from our study confirm the findings from the abovementioned studies and more importantly include data from newer DAAs (sofosbuvir/velpatasvir/voxilaprevir and glecaprevir/pibrentasvir).

Our results should be viewed in the context that we used the Roche COBAS TaqMan HCV assay, which has an LLOQ of 15 IU/mL. The more sensitive Abbot RealTime has an LLOQ of 12, which may lead to a higher frequency of residual HCV RNA detection, though clinical management would remain the same [17, 18].

A major strength of this study was the number of patients included, and the major limitation was its retrospective design.

## 5. Conclusion

Low positive EOT VL does not negatively affect SVR12 and SVR24 in the DAA era, hence should not lead to extending treatment duration. We propose that EOT VL measurements should be avoided in most patients due to the limited clinical utility of the result.

## Figures and Tables

**Table 1:** Demographic, baseline lab, and treatment characteristics in EOT positive viral load patients.

Patient	Gender	Race	Age	BMI (kg/m^2^)	INR	Albumin (g/dL)	Total bilirubin (mg/dL)	Platelet (x 1,000/mm^3^)	ALT (IU/L)	Baseline viral load (IU/mL)	Week 4 viral load (IU/mL)	EOT viral load (IU/mL)	Genotype	Fibrosis level (stage)	Treatment regimen	Previous treatment	SVR 12/24
1	F	Caucasian	70	30	—	3.6	1	312	53	3,715,090	Neg	62	1a	2	SOF + SIM	Peg-IFN + RBV	Y/Y
2	F	Caucasian	62	27	1.1	4.4	0.6	218	77	3,895,292	<15	235	2b	—	SOF + RBV	—	Y/Y
3	F	Caucasian	64	32	—	3.9	0.31	189	27	3,200,000	Neg	26	1b	0	SOF + LED	—	Y/Y
4	F	Black	71	27	—	3.6	0.4	357	35	971,341	Neg	<15	1a	—	SOF + LED	—	Y/Y
5^*∗*^	M	Caucasian	53	23	—	4	0.5	183	39	2,108,530	Neg	Neg	1a	—	SOF + LED	Peg-IFN + RBV	Y/Y
6	M	Caucasian	29	29	—	0.3	0.3	340	118	1,540,000	120	28	1a	0	SOF + LED	—	Y/Y
7	M	Caucasian	68	30	1.2	1.1	1.1	86	73	3,001,000	640	62	1a	4	SOF + LED	—	Y/Y
8	F	Caucasian	52	—	1.1	1.1	1.1	94	82	2,300,000	206	18	1a	4	SOF + VEL + VOX	Peg-IFN + RBV SOF + LED	Y/Y
9	F	Caucasian	62	—	0.9	0.4	0.4	239	157	2,108,530	128	14	1a	2	GLE + PIB	—	Y/Y

^*∗*^Patients had a negative EOT viral load, but 23 IU/mL at week four after EOT. Abbreviations: SOF, sofosbuvir; SIM, simeprevir; peg-IFN, pegylated interferon; RBV, ribavirin; LED, ledipasvir; VEL, velpatasvir; VOX, voxilaprevir; GLE, glecaprevir; PIB, pibrentasavir.

## Data Availability

Data are available on request.
